# Supplementary material for: Resolving the full spectrum of human genome variation using Linked-Reads
Source: Genome Res. 2019 Apr;29(4):635–45. doi: 10.1101/gr.234443.118 (PMC6442396; doi:10.1101/gr.234443.118)
Supplement: Supplemental Material [file supp_gr.234443.118_Supplemental_longranger-2.2.zip › longranger-2.2.tar.bz2!/lr2.2-public/lib/stan/cmdstan/stan/doc/api/html/structstan_1_1math_1_1coupled__ode__system_3_01_f_00_01stan_1_1agrad_1_1var_00_01stan_1_1agrad_1_1var_01_4-members.html]

Stan: Member List


|  |  |
| --- | --- |
|  | Stan  2.5.0  probability, sampling & optimization |


- Main Page
- Related Pages
- Namespaces
- Classes
- Files

- Class List
- Class Index
- Class Hierarchy
- Class Members

All Classes Namespaces Files Functions Variables Typedefs Enumerations Enumerator Friends Macros Pages

- stan
- math
- coupled\_ode\_system< F, stan::agrad::var, stan::agrad::var >

stan::math::coupled\_ode\_system< F, stan::agrad::var, stan::agrad::var > Member List

This is the complete list of members for stan::math::coupled\_ode\_system< F, stan::agrad::var, stan::agrad::var >, including all inherited members.

|  |  |  |
| --- | --- | --- |
| coupled\_ode\_system(const F &f, const std::vector< stan::agrad::var > &y0, const std::vector< stan::agrad::var > &theta, const std::vector< double > &x, const std::vector< int > &x\_int, std::ostream \*msgs) | stan::math::coupled\_ode\_system< F, stan::agrad::var, stan::agrad::var > | inline |
| decouple\_states(const std::vector< std::vector< double > > &y) | stan::math::coupled\_ode\_system< F, stan::agrad::var, stan::agrad::var > | inline |
| f\_ | stan::math::coupled\_ode\_system< F, stan::agrad::var, stan::agrad::var > |  |
| initial\_state() | stan::math::coupled\_ode\_system< F, stan::agrad::var, stan::agrad::var > | inline |
| M\_ | stan::math::coupled\_ode\_system< F, stan::agrad::var, stan::agrad::var > |  |
| msgs\_ | stan::math::coupled\_ode\_system< F, stan::agrad::var, stan::agrad::var > |  |
| N\_ | stan::math::coupled\_ode\_system< F, stan::agrad::var, stan::agrad::var > |  |
| operator()(const std::vector< double > &y, std::vector< double > &dy\_dt, double t) | stan::math::coupled\_ode\_system< F, stan::agrad::var, stan::agrad::var > | inline |
| size() const | stan::math::coupled\_ode\_system< F, stan::agrad::var, stan::agrad::var > | inline |
| size\_ | stan::math::coupled\_ode\_system< F, stan::agrad::var, stan::agrad::var > |  |
| theta\_ | stan::math::coupled\_ode\_system< F, stan::agrad::var, stan::agrad::var > |  |
| theta\_dbl\_ | stan::math::coupled\_ode\_system< F, stan::agrad::var, stan::agrad::var > |  |
| x\_ | stan::math::coupled\_ode\_system< F, stan::agrad::var, stan::agrad::var > |  |
| x\_int\_ | stan::math::coupled\_ode\_system< F, stan::agrad::var, stan::agrad::var > |  |
| y0\_ | stan::math::coupled\_ode\_system< F, stan::agrad::var, stan::agrad::var > |  |
| y0\_dbl\_ | stan::math::coupled\_ode\_system< F, stan::agrad::var, stan::agrad::var > |  |


---

[ Stan Home Page ]

*© 2011–2014,
Stan Development Team.*
